# A prospective study of dietary and supplemental zinc intake and risk of type 2 diabetes depending on genetic variation in *SLC30A8*

**DOI:** 10.1186/s12263-017-0586-y

**Published:** 2017-10-30

**Authors:** Isabel Drake, George Hindy, Ulrika Ericson, Marju Orho-Melander

**Affiliations:** Diabetes and Cardiovascular Disease – Genetic Epidemiology, Lund University Diabetes Centre, Department of Clinical Sciences in Malmö, Lund University, Clinical Research Center 60:13, Jan Waldenströms gata 35, SE-205 02 Malmö, Sweden

**Keywords:** Gene-nutrient interaction, Zinc, Solute carrier family 30 member 8 gene, Single nucleotide polymorphism, Cohort, Body mass index

## Abstract

**Background:**

The solute carrier family 30 member 8 gene (*SLC30A8*) encodes a zinc transporter in the pancreatic beta cells and the major C-allele of a missense variant (rs13266634; C/T; R325W) in *SLC30A8* is associated with an increased risk of type 2 diabetes (T2D). We hypothesized that the association between zinc intake and T2D may be modified by the *SLC30A8* genotype.

**Results:**

We carried out a prospective study among subjects with no history cardio-metabolic diseases in the Malmö Diet and Cancer Study cohort (*N* = 26,132, 38% men; 86% with genotype data). Zinc intake was assessed using a diet questionnaire and food record. During a median follow-up of 19 years, 3676 T2D cases occurred. A BMI-stratified Cox proportional hazards regression model with attained age as the time scale was used to model the association between total and dietary zinc intake, zinc supplement use, zinc to iron ratio, and risk of T2D adjusting for putative confounding factors.

The median total zinc intake was 11.4 mg/day, and the median dietary zinc intake was 10.7 mg/day. Zinc supplement users (17%) had a median total zinc intake of 22.4 mg/day. Dietary zinc intake was associated with increased risk of T2D (*P*_trend_ < 0.0001). In contrast, we observed a lower risk of T2D among zinc supplement users (HR = 0.79, 95% CI 0.70–0.89). The *SLC30A8* CC genotype was associated with a higher risk of T2D (HR = 1.16, 95% CI 1.07–1.24), and the effect was stronger among subjects with higher BMI (*P*_interaction_ = 0.007). We observed no significant modification of the zinc-T2D associations by *SLC30A8* genotype. However, a three-way interaction between *SLC30A8* genotype, BMI, and zinc to iron ratio was observed (*P*_interaction_ = 0.007). A high zinc to iron ratio conferred a protective associated effect on T2D risk among obese subjects, and the effect was significantly more pronounced among T-allele carriers.

**Conclusions:**

Zinc supplementation and a high zinc to iron intake ratio may lower the risk of T2D, but these associations could be modified by obesity and the *SLC30A8* genotype. The findings implicate that when considering zinc supplementation for T2D prevention, both obesity status and *SLC30A8* genotype may need to be accounted for.

**Electronic supplementary material:**

The online version of this article (10.1186/s12263-017-0586-y) contains supplementary material, which is available to authorized users.

## Background

Prevalence of type 2 diabetes (T2D) is increasing worldwide attributable to aging populations and increasing prevalence of obesity due to obesogenic environmental changes [1]. While both genetic and environmental factors are considered to increase risk, the most important targets for prevention of obesity and T2D are modifiable factors including diet and physical activity [2]. Zinc is an essential trace element with both structural and functional roles in many cellular proteins and enzymes. It plays a vital role in β-cell physiology and insulin action [3, 4]. In animal studies, zinc intake has been suggested to be protective of T2D [5]. Two studies in women examining the association between zinc intake and prospective risk of T2D indicated that high dietary zinc intake and/or a high zinc to iron ratio was associated with a lower risk of T2D [6, 7]. Placebo-controlled randomized trials have found that zinc supplementation modestly reduces fasting glucose and hemoglobin A1c (HbA1c), particularly in patients with T2D [8]. Due to limited number of controlled trials, there is to date no overall strong evidence supporting that zinc supplementation may lower the risk of T2D in humans [9]. One of the most consistently replicated single-nucleotide variants increasing the risk of T2D is rs13266634 (C/T) in the *SLC30A8* gene that encodes zinc transporter 8, which is mainly expressed in the pancreatic β-cells. The major C-allele of rs13266634 is associated with a lower early insulin response to glucose and a higher risk of T2D [10–14]. As the rs13266634 is a non-synonymous variant, the effect of zinc on T2D risk could plausibly vary across this genotype. Indeed, a recent case-control study observed an interaction between plasma zinc levels and rs13266634 on T2D risk [15]. However, prospective studies investigating whether the association between zinc intake and risk of T2D differ depending on rs13266634 genotype are lacking. Given the biological plausibility for a potential interaction between zinc intake and *SLC30A8* rs13266634 on T2D risk, and the scarcity of large prospective studies examining the role of zinc intake in relation to T2D risk, we wanted to examine these associations in a large population-based prospective cohort of middle-aged Swedish men and women. As supplemental zinc is more bioavailable than zinc from food sources [16], and as iron has been shown to inhibit the absorption of zinc [17, 18], we set out to examine zinc intake from both food sources and supplements, the zinc to iron ratio in relation to T2D, and particularly, potential effect modification by the *SLC30A8* rs13266634 genotype.

## Methods

### Study population

The Malmö Diet and Cancer Study (MDCS) is a population-based prospective cohort set in the south of Sweden [19]. Baseline examinations were carried out between March 1991 and October 1996. The source population included all persons living in the city of Malmö born between 1926 and 1945 and with sufficient Swedish reading and writing skills. With a participation rate of 40%, 28,098 participants completed all baseline examinations [20]. From this study population, we excluded subjects with a history of prevalent diabetes mellitus or cardiovascular disease, resulting in a total study population of 26,132. A flow chart of the analytical study population, including information on covariates with missing data, is shown in Additional file 1: Figure S1.

### Follow-up and case ascertainment

All subjects were followed from entry into the study until their date of diabetes mellitus diagnosis, date of death from any cause, emigration, or end of follow-up (31 December 2014), whichever came first. Information on vital and emigration status of the study participants was obtained from the Swedish Cause of Death Registry and the Swedish Tax Agency. Diabetes status at baseline and during follow-up and information on date of diabetes diagnosis were identified from seven registers as well as baseline and re-examination screenings of the MDCS and the Malmö Preventive Project [21]. The National Diabetes Register [22] and the regional Diabetes 2000 Register [23] required a proven diagnosis by a physician at the hospital based on international standards for diagnosis (i.e., fasting plasma glucose concentration ≥ 7.0 mmol/l measured twice). For cases not diagnosed at a hospital, the local HbA1c register from the Department of Clinical Chemistry, Skåne University Hospital, Malmö, was used [24, 25]. Other registries used to identify diabetes cases included the National Patient Register, the Swedish Cause of Death Register (ICD10 codes E10-E14 and O244-O249), and the Prescribed Drug Register (ATC code A10). The different sources of case ascertainment were overlapping. Of the total 3831 diabetes diagnoses identified during follow-up, 2397 cases were captured by the National Diabetes Register. Information on diabetes type was lacking for most of the cases (54%). For subjects where type was specified, we censored cases of type 1 diabetes (*n* = 136), LADA (*n* = 9), secondary diabetes (*n* = 1), and other (*n* = 9) at the date of diagnosis. For the remaining cases (*n* = 3676), given the age distribution at diagnosis, we assumed that they were T2D.

### Genotyping

Genotyping was performed at the Clinical Research Centre, Malmö, Sweden, using Sequenom MassARRAY iPLEX (Sequenom, San Diego, CA, USA) according to the manufacturers’ instructions. The concordance rate was >99% in a subset of 5500 samples which were additionally genotyped using Human Omni Express Exome Bead Chip Kit (Illumina, San Diego, CA, USA). Genotyping success rate was 97.3%. No deviation from the Hardy-Weinberg equilibrium was observed (*P* = 0.82). In total, 20,929 subjects in the current study population had genotype data for the *SLC30A8* rs13266634 variant.

### Dietary variables

Dietary assessment was conducted using a modified diet history method including a 168-item diet questionnaire (using exact frequencies and pictures to assess portion sizes), a 7-day food record (in which descriptions of prepared meals, nutrient supplements, and cold beverages were collected), and a 1-h dietary interview. Data on the validity [26, 27] and reproducibility [28] of the method have been published. Energy and nutrient intakes were computed using the MDC Food and Nutrient Database, mainly originating from the PC Kost2-93 food database of the Swedish National Food Administration. Use of dietary supplements (including type and amount) was registered in the 7-day food record and categorized as any diet supplement use (yes/no) and zinc supplement use (yes/no). Total zinc and iron intakes (mg/day) were estimated by combining intake from both food sources and supplements. The zinc to iron ratio was calculated as the ratio of total energy-adjusted zinc to energy-adjusted total iron intake. Other dietary variables included in the main analyses were total energy intake, alcohol, fiber, fruit and vegetables, processed meat, sugar-sweetened beverages, and coffee intake. Further, in sensitivity analysis, we examined additional adjustments for saturated fatty acids, fish and shellfish, red meat, total protein (as percentage of total energy intake), iron, and calcium. Food and nutrient intakes were energy-adjusted using the residual method [29] and participants ranked into quintiles to reduce the influence of outliers and handle highly skewed intake levels. Participants were classified as misreporters of energy intake if the ratio of energy intake to basal metabolic rate was outside the 95% confidence interval of the calculated physical activity level [30]. Participants with potentially unstable food habits over time were identified using the questionnaire item “Have you previously changed your food habits substantially due to illness or other reason?” (yes/no) [31]. Season of dietary data collection (January–March, April–June, July–September, October–December) was adjusted for as a categorical variable to account for seasonal variation in reported food intakes. Calendar year of study entry was adjusted for as a categorical variable to account for the recruitment of slightly older individuals during the last 2 years of baseline examinations and to account for a minor change in coding routines for the dietary assessment in September 1994 [32].

### Lifestyle and other variables

Age and sex were determined by the participants’ Swedish personal identification numbers. Nurses measured height (cm) and weight (kg) with subjects wearing light indoor clothing with no shoes. Body mass index (BMI) was calculated as weight divided by height squared (kg/m^2^). BMI was classified as normal-weight (< 25 kg/m^2^), overweight (25–29.9 kg/m^2^), and obese (≥ 30 kg/m^2^). Information on socioeconomic and lifestyle factors was collected from the MDC baseline questionnaire [33]. Educational level was categorized as elementary, primary and secondary, upper secondary, further education without a degree, or university/college degree. Smoking status was defined as never, former, or current (including irregular smokers). Leisure time physical activity was estimated based on an adaptation of the Minnesota Leisure Time Physical Activity Instrument [34, 35]. Participants estimated the number of minutes per week spent on 17 different physical activities for each of the four seasons, and a score was calculated by multiplying an intensity factor with the duration of each activity. The score was categorized into sex-specific quartiles.

### Statistical analysis

Baseline characteristics across quintiles of energy-adjusted total, dietary, zinc to iron ratio, and zinc supplement use (yes/no) were examined. Mean values (standard deviation, SD) of continuous variables were calculated for each category of zinc intake. For skewed dietary variables, geometric mean values (95% confidence intervals, CIs) were calculated. Further, we examined the baseline characteristics by T2D status and *SLC30A8* genotype. We used a BMI-stratified Cox proportional hazards regression models with attained age as the underlying time metric to estimate hazard ratios (HRs) and 95% CIs for the association between the different measures of zinc intake in relation to risk of incident T2D. We examined the non-linear association between total zinc intake and risk of T2D by fitting a restricted cubic spline with three knots chosen according to Harrell’s recommended percentiles to a Cox proportional hazards regression model using a continuous variable for total zinc intake (mg/day). Extreme intakes (> 50 mg/day) were excluded from this analysis. The main multivariable model included both dietary (total energy intake, season of dietary data collection, dietary fiber, alcohol consumption, fruit and vegetables, processed meats, sugar-sweetened beverages, coffee intake, and any diet supplement use) and non-dietary covariates (age, calendar year of study entry, BMI, sex, educational level, smoking status, leisure time physical activity). Sensitivity analyses included exclusion of subjects classified as energy misreporters and those reporting substantial dietary changes in the past. For all presented Cox models, the proportional hazards (PH) assumption was fulfilled as determined by the Schoenfeld test. Since including BMI as a covariate violated the PH assumption, we present the BMI-stratified Cox model for the main analyses and further examined potential interactions with BMI. In the MDCS, sufficient statistical power to examine dietary hypotheses (i.e., 80% and *α* = 0.05) assuming a case-control design with three controls per case and a validity coefficient of the dietary variable of 0.6 was reached when more than 283 cases had accumulated. Statistical power to detect an interaction between rs13266634 genotype with zinc intake was examined using Quanto version 1.2.4 (http://biostats.usc.edu/software). In this study, we had 80% power to detect an interaction odds ratio of at least 1.30 for T2D. Stata SE/14.2 for Mac (StataCorp, College Station, TX) was used for all statistical analyses. All reported tests were two-sided, and *P* values < 0.05 were considered statistically significant.

## Results

### Baseline characteristics of the study population

During a median follow-up time of 19 years, we identified 3676 incident cases of T2D. The mean age at diagnosis was 69.1 years (SD = 8.1). In total, 4417 subjects (17%) reported use of supplements containing zinc. Subjects with high dietary zinc intake were more likely to be younger, be male, and have higher BMI and educational level and were less likely to be current smokers compared to subjects with low dietary zinc intake. Zinc supplement users were more likely to be female, older, and current smokers and to have lower educational level compared to non-users (Table 1). Baseline characteristics of the study population by T2D status and *SLC30A8* rs13266634 genotype are shown in Additional file 1: Tables S1 and S2, respectively.

**Table 1:** Participant characteristics by quintiles (Q) of energy-adjusted total zinc, dietary zinc, zinc to iron ratio, and zinc supplement use (yes/no) in the Malmö Diet and Cancer Study at baseline (1991–1996)

	Total zinc intake					Dietary zinc intake					Zinc to iron ratio					Zinc supplement use	
	Q1	Q2	Q3	Q4	Q5	Q1	Q2	Q3	Q4	Q5	Q1	Q2	Q3	Q4	Q5	No	Yes
Number of subjects	5234	5228	5223	5221	5226	5234	5231	5224	5219	5224	5223	5227	5225	5228	5229	21,715	4417
Total zinc (mg/day)	8.6 (2.1)	10.3 (2.3)	11.5 (2.6)	13.1 (3.0)	21.4 (7.6)	10.5 (5.7)	12.1 (5.7)	12.8 (5.6)	13.9 (6.0)	15.5 (6.1)	12.6 (4.6)	12.3 (3.0)	11.5 (4.1)	15.1 (7.8)	13.3 (8.3)	11.2 (3.4)	21.7 (8.3)
Total iron (mg/day)	13.7 (4.4)	14.9 (4.9)	15.8 (5.2)	16.7 (5.9)	16.2 (5.8)	17.1 (14.5)	17.6 (11.1)	18.3 (12.7)	18.5 (10.9)	19.0 (10.7)	17.7 (5.4)	20.5 (22.3)	15.7 (9.3)	19.4 (8.9)	17.2 (5.3)	16.4 (11.6)	26.3 (11.2)
Dietary zinc (mg/day)	8.6 (2.1)	10.2 (2.3)	11.5 (2.6)	13.0 (3.1)	12.5 (4.1)	8.7 (2.3)	10.2 (2.5)	11.1 (2.8)	12.0 (2.9)	13.8 (3.8)	11.9 (3.5)	12.1 (3.0)	10.9 (3.1)	10.3 (3.1)	10.5 (3.5)	11.2 (3.4)	10.9 (3.1)
Zinc supplement (mg/day) among users	0.8 (0.5)	1.0 (0.6)	1.2 (0.7)	1.6 (1.1)	12.6 (7.1)	10.7 (8.5)	11.0 (7.4)	10.6 (7.5)	10.9 (8.1)	10.5 (7.1)	7.3 (7.5)	1.5 (1.5)	7.8 (5.6)	13.3 (4.3)	13.2 (10.2)	–	10.8 (7.7)
Demography and lifestyle																	
Age (years)	59.3 (7.8)	58.2 (7.6)	57.5 (7.5)	56.6 (7.2)	57.5 (7.6)	59.4 (7.9)	58.7 (7.7)	57.9 (7.6)	57.0 (7.4)	56.2 (7.0)	57.5 (7.4)	57.0 (7.4)	57.7 (7.6)	58.7 (7.8)	58.3 (7.8)	57.7 (7.6)	58.4 (7.8)
Male sex (%)	34.1	36.1	40.2	43.4	35.1	35.7	36.1	36.9	37.7	41.6	44.0	38.9	35.6	32.0	38.5	39.9	27.2
BMI (kg/m^2^)	25.4 (3.9)	25.5 (3.8)	25.8 (3.9)	25.9 (4.0)	25.4 (3.9)	25.2 (3.8)	25.4 (3.8)	25.5 (3.8)	25.7 (3.9)	26.1 (4.0)	25.7 (3.9)	25.8 (4.0)	25.6 (3.9)	25.3 (3.8)	25.5 (3.9)	25.8 (3.9)	24.9 (3.7)
< 25 (%)	49.8	49.6	46.9	45.3	51.1	51.7	50.8	49.6	47.7	42.7	47.0	46.5	48.0	51.6	49.4	46.7	57.3
25–29.9 (%)	38.4	39.2	39.4	40.7	37.8	37.8	38.0	38.1	39.4	42.2	40.5	39.3	39.2	37.3	39.3	40.1	34.1
≥ 30 (%)	11.8	11.3	13.7	14.0	11.2	10.5	11.2	12.2	13.0	15.1	12.4	14.2	12.8	11.2	11.4	13.2	8.5
High physical activity (%)	24.3	25.3	24.6	25.0	25.9	25.1	26.2	24.6	25.6	23.6	24.9	25.2	24.6	24.6	25.7	24.5	27.7
Current smoker (%)	31.5	27.8	28.9	27.5	26.7	31.2	27.6	27.8	27.1	28.6	27.1	30.0	30.4	27.4	27.5	29.3	24.3
High educational level (%)	11.8	13.9	14.0	15.5	17.9	12.7	13.8	14.9	15.6	16.0	14.6	15.3	13.9	14.2	15.0	13.7	19.1
Diet																	
Total energy (kcal)	2056 (2041–2072)	2132 (2116–2148)	2190 (2173–2206)	2229 (2212–2246)	2172 (2156–2189)	2142 (2126–2158)	2164 (2148–2180)	2162 (2146–2179)	2166 (2150–2182)	2141 (2124–2159)	2210 (2192–2227)	2188 (2171–2205)	2150 (2133–2166)	2087 (2072–2102)	2143 (2127–2159)	2161 (2153–2169)	2126 (2109–2142)
Alcohol (g/day)	8.0 (7.7–8.3)	7.7 (7.4–8.0)	7.8 (7.6–8.1)	7.9 (7.6–8.1)	7.5 (7.3–7.8)	8.5 (8.2–8.9)	7.7 (7.4–8.0)	7.7 (7.5–8.0)	7.6 (7.4–7.9)	7.3 (7.1–7.6)	7.8 (7.5–8.1)	7.7 (7.4–8.0)	7.4 (7.1–7.6)	7.7 (7.5–8.0)	8.3 (8.0–8.6)	7.8 (7.7–8.0)	7.5 (7.2–7.7)
Dietary fiber (g/day)	16.4 (16.3–16.6)	18.2 (18.0–18.3)	19.2 (19.1–19.4)	20.3 (20.1–20.5)	20.4 (20.2–20.6)	17.0 (16.8–17.1)	18.4 (18.2–18.5)	19.0 (18.8–19.2)	19.7 (19.6–19.9)	20.4 (20.2–20.6)	20.2 (20.0–20.4)	19.2 (19.0–19.4)	18.0 (17.8–18.1)	18.2 (18.0–18.3)	18.8 (18.6–18.9)	18.6 (18.6–18.7)	19.9 (19.7–20.1)
Processed meat (g/day)	31.2 (30.5–32.0)	37.2 (36.4–38.1)	39.8 (38.9–40.7)	41.9 (41.0–42.9)	37.4 (36.5–38.3)	31.8 (31.0–32.6)	36.7 (35.9–37.6)	38.4 (37.5–39.3)	39.4 (38.5–40.3)	41.0 (40.0–41.9)	41.2 (40.2–42.2)	39.1 (38.2–40.1)	36.8 (36.0–37.7)	34.2 (33.4–35.0)	35.9 (35.1–36.7)	38.2 (37.7–38.6)	33.5 (32.6–34.4)
Sugar-sweetened beverages (g/day)	89.0 (84.8–93.4)	71.7 (68.2–75.4)	69.4 (66.1–72.9)	63.9 (60.7–67.3)	60.7 (57.5–64.2)	95.2 (90.8–99.9)	73.5 (69.9–77.3)	66.6 (63.4–70.0)	63.1 (60.0–66.4)	56.8 (53.7–60.1)	66.6 (63.1–70.2)	68.7 (65.3–72.3)	78.8 (75.0–82.8)	71.9 (68.3–75.7)	69.2 (65.9–72.8)	72.6 (70.8–74.4)	63.3 (59.7–67.2)
Fruit and vegetables (g/day)	295.0 (290.3–299.8)	322.6 (318.2–327.0)	334.1 (329.4–338.8)	349.4 (344.6–354.3)	361.5 (356.5–366.5)	299.3 (294.6–304.1)	328.1 (323.5–332.8)	332.1 (327.5–336.7)	344.5 (339.9–349.2)	357.4 (352.4–362.5)	340.4 (335.5–345.3)	334.5 (329.7–339.4)	322.9 (318.2–327.7)	330.4 (325.8–335.1)	330.6 (325.8–335.3)	325.8 (323.5–328.2)	362.2 (357.0–367.4)
Coffee (g/day)	405.1 (396.3–414.1)	411.0 (402.4–419.8)	435.0 (425.9–444.3)	437.4 (427.8–447.2)	418.5 (409.3–427.9)	404.6 (395.8–413.6)	407.7 (399.1–416.5)	415.9 (407.1–424.8)	432.4 (423.1–441.8)	447.2 (437.2–457.4)	426.0 (416.9–435.3)	438.1 (428.5–448.0)	424.1 (414.9–433.4)	401.3 (392.7–410.1)	417.3 (408.4–426.4)	427.7 (423.2–432.3)	390.2 (381.0–400.0)
Diet supplement user (%)	62.6	63.3	61.1	62.2	87.4	68.0	68.2	68.3	65.5	66.6	63.6	65.0	63.4	76.1	68.6	60.7	100.0

Continuous variables expressed as mean (standard deviation, SD) and categorical variables as percentages. Continuous dietary variables other than zinc intakes are expressed as geometric means (95% confidence interval)

### Zinc intake and risk of T2D

**Fig. 1:**
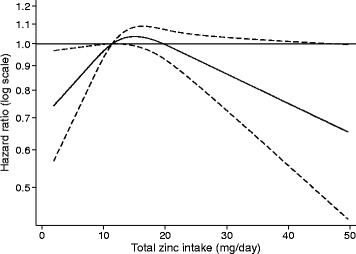
Restricted cubic spline representing the association between total zinc intake and risk of type 2 diabetes in the Malmö Diet and Cancer Study (*n* = 26,132). Median total zinc intake (11.4 mg/day) was used as the reference point. Bold line represents hazard ratio (HR), and dotted lines represent the 95% confidence interval. The spline was fitted using a BMI-stratified Cox proportional hazards model with attained age as the time metric and adjusting for dietary and non-dietary confounders

In multivariable analyses, we observed that total zinc intake was non-linearly associated with risk of T2D (Fig. 1; Table 2). The increased risk associated with total zinc intake appeared to be driven by a linear positive association between dietary zinc intake and T2D (Table 2). This association was attenuated, but nominally significant, after adjusting for both dietary and non-dietary confounders, and excluding potential energy misreporters and subjects with unstable food habits (Q5 versus Q1: HR = 1.27, 95% CI 1.06–1.51; *P*_trend_ = 0.001). With additional adjustment for total protein intake, total iron intake, and intake of red meat, the association was further attenuated and no longer significant (Q5 versus Q1: HR = 1.07, 95% CI 0.88–1.30; *P*_trend_ = 0.42; data not tabulated). Zinc supplement use was found to associate with a lower risk of T2D (HR = 0.79, 95% CI 0.70–0.89), and the inverse association remained significant in multivariable analyses (Table 2). We further observed that a high zinc to iron ratio was associated with a marginally lower risk of T2D (Q5 versus Q1: HR = 0.91 (95% CI 0.81–1.02; *P*_trend_ = 0.02), although the association was attenuated in multivariable and sensitivity analyses (Table 2). Additional adjustment for total iron intake did not affect the observed association.

**Table 2:** Hazard ratios (HRs) and 95% confidence intervals (CIs) for type 2 diabetes (T2D) by quintiles (Q) of energy-adjusted total and dietary zinc intake, zinc to iron ratio, and zinc supplement use (yes/no) in the Malmö Diet and Cancer Study (1991–2014)

	Total zinc intake					
	Q1	Q2	Q3	Q4	Q5	*P*_trend_
Number of subjects (T2D cases)	5234 (638)	5228 (719)	5223 (789)	5221 (832)	5226 (698)	
Person-years	88,923	91,333	90,929	91,301	93,281	
Model 1	1.00 (ref)	1.10 (0.99–1.23)	1.22 (1.10–1.35)	1.29 (1.16–1.43)	1.07 (0.96–1.19)	0.017
Model 2	1.00 (ref)	1.14 (1.00–1.29)	1.19 (1.06–1.35)	1.25 (1.11–1.41)	1.14 (1.01–1.29)	0.011
Model 3	1.00 (ref)	1.17 (1.03–1.32)	1.23 (1.08–1.39)	1.29 (1.14–1.47)	1.16 (1.02–1.32)	0.008
Model 4	1.00 (ref)	1.09 (0.92–1.29)	1.11 (0.94–1.32)	1.26 (1.06–1.49)	1.05 (0.88–1.25)	0.212
	Dietary zinc intake					
	Q1	Q2	Q3	Q4	Q5	*P*_trend_
Number of subjects (T2D cases)	5234 (617)	5231 (674)	5224 (705)	5219 (808)	5224 (872)	
Person-years	89,374	91,085	91,528	92,067	91,714	
Model 1	1.00 (ref)	1.08 (0.97–1.20)	1.14 (1.02–1.27)	1.31 (1.18–1.46)	1.43 (1.29–1.58)	< 0.0001
Model 2	1.00 (ref)	1.10 (0.97–1.25)	1.15 (1.01–1.30)	1.25 (1.11–1.42)	1.31 (1.16–1.48)	< 0.0001
Model 3	1.00 (ref)	1.14 (1.00–1.29)	1.19 (1.05–1.35)	1.32 (1.16–1.49)	1.39 (1.22–1.57)	< 0.0001
Model 4	1.00 (ref)	1.05 (0.89–1.25)	1.12 (0.94–1.32)	1.26 (1.06–1.49)	1.27 (1.06–1.51)	0.001
	Zinc to iron ratio					
	Q1	Q2	Q3	Q4	Q5	*P*_trend_
Number of subjects (T2D cases)	5223 (782)	5227 (801)	5225 (740)	5228 (656)	5229 (697)	
Person-years	90,860	90,977	90,965	91,825	91,141	
Model 1	1.00 (ref)	1.06 (0.96–1.17)	0.98 (0.89–1.08)	0.86 (0.77–0.95)	0.90 (0.81–1.00)	< 0.0001
Model 2	1.00 (ref)	1.02 (0.91–1.14)	0.92 (0.82–1.03)	0.91 (0.81–1.03)	0.91 (0.81–1.02)	0.027
Model 3	1.00 (ref)	1.01 (0.90–1.14)	0.91 (0.82–1.03)	0.90 (0.80–1.02)	0.91 (0.81–1.02)	0.023
Model 4	1.00 (ref)	1.02 (0.87–1.20)	0.96 (0.82–1.13)	0.92 (0.78–1.08)	0.91 (0.77–1.07)	0.110
	Zinc supplement use					
	No	Yes				
Number of subjects (T2D cases)	21,715 (3225)	4417 (451)				
Person-years	376,020	79,748				
Model 1	1.00 (ref)	0.68 (0.62–0.75)				
Model 2	1.00 (ref)	0.83 (0.74–0.92)				
Model 3	1.00 (ref)	0.79 (0.70–0.89)				
Model 4	1.00 (ref)	0.83 (0.71–0.98)				

*Model 1* Cox proportional hazards model with attained age as the time metric and adjusted for sex; *Model 2* BMI-stratified Cox proportional hazards model with attained age as time metric and adjusted for sex, calendar year of study entry, educational level, smoking status, leisure-time physical activity, season of dietary data collection, and total energy intake; *Model 3* as model 2 with additional adjustment for alcohol consumption, dietary fiber, fruit and vegetables, processed meat, sugar-sweetened beverages, coffee intake and diet supplement use (any); *Model 4* as model 3 with exclusion of past food habit changers and potential energy misreporters (n excluded = 9204

### *SLC30A8* rs13266634, zinc intake, and risk of type 2 diabetes

**Fig. 2:**
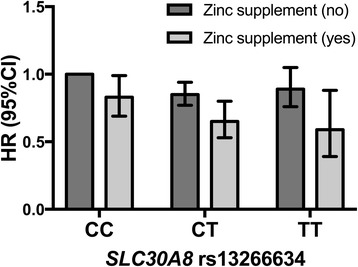
Zinc supplement use and risk of T2D across *SLC30A8* rs13266634 genotype in the MDCS (*n* = 20,929). Hazard ratios (HR) from a Cox proportional hazards model adjusting for dietary and non-dietary confounders. *P*_interaction_ (genotype × zinc supplement use) = 0.32

The CC genotype of *SLC30A8* rs13266634, compared to CT/TT, was associated with a higher risk of T2D (HR = 1.16, 95% CI 1.07–1.24, *P* = 9.6 × 10^−5^) (Additional file 1: Figure S2). We observed no significant multiplicative interactions between quintiles of total zinc intake (*P*_interaction_ = 0.83), dietary zinc intake (*P*_interaction_ = 0.53), total zinc to iron ratio (*P*_interaction_ = 0.32), or zinc supplement use (yes/no; *P*_interaction_ = 0.44) and the *SLC30A8* genotype in the fully adjusted model including both dietary and non-dietary factors. However, the lowest risk was observed among the group of zinc supplement users with the TT genotype (HR = 0.59, 95% CI 0.39–0.88) compared to non-supplement users with the CC genotype (Fig. 2). Further, a high zinc to iron ratio was non-significantly associated with lower risk of T2D among CC/CT genotypes while a clear protective association was observed among the smaller group of TT carriers (*P*_trend_ = 0.009; data not tabulated).

### *SLC30A8* rs13266634, BMI, zinc intake, and risk of type 2 diabetes

**Fig. 3:**
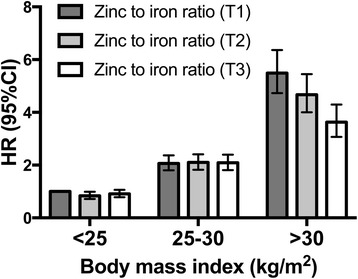
Tertiles of zinc to iron ratio and risk of T2D across categories of BMI in the MDCS (*n* = 26,132). Hazard ratios (HR) from a Cox proportional hazards model adjusting for dietary and non-dietary confounders. *P*_interaction_ (zinc to iron ratio × BMI) = 0.005

**Fig. 4:**
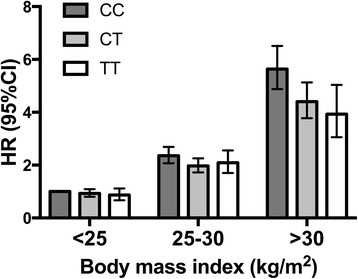
*SLC30A8* rs13266634 genotype and risk of T2D across categories of BMI in the MDCS (*n* = 20,929). Hazard ratios (HR) from a Cox proportional hazards model with attained age as the time scale with adjustment for non-dietary confounders, total energy intake, and diet supplement use (any). *P*_interaction_ (genotype × BMI) = 0.007

**Fig. 5:**
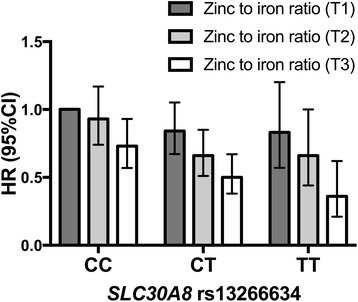
Tertiles of zinc to iron ratio and risk of T2D across *SLC30A8* rs13266634 genotype among obese subjects in the MDCS (*n* = 2550). Hazard ratios (HR) from a Cox proportional hazards model adjusting for dietary and non-dietary confounders. *P*_three-way interaction_ (zinc to iron ratio × BMI × genotype) = 0.007

In a non-stratified Cox model including BMI as a continuous covariate and adjusting for dietary and non-dietary factors (same as those presented in model 3, Table 2), we observed no interaction between BMI and total zinc intake (*P*_interaction_ = 0.08), dietary zinc intake (*P*_interaction_ = 0.32), or zinc supplement use (*P*_interaction_ = 0.15) on the risk of T2D. However, we did observe a significant interaction between BMI and the zinc to iron ratio (*P*_interaction_ = 0.005; Fig. 3). The zinc to iron ratio was only significantly associated with lower risk of T2D among subjects with high BMI. The HR for the highest tertile of zinc to iron ratio compared to the lowest tertile among obese subjects (BMI >30 kg/m^2^) was 0.70 (95% CI 0.60–0.82; *P*_trend_ = 1.4 × 10^−5^). We further found that the T2D risk-increasing CC genotype was not associated with an increased risk among normal-weight subjects (HR = 1.09, 95% CI 0.94–1.26). Among overweight (HR = 1.15, 95% CI 1.04–1.28) and obese (HR = 1.31, 95% CI 1.14–1.50) subjects, the CC genotype was associated with a higher risk of T2D. There was a significant interaction between the *SLC30A8* genotype and BMI after adjustment for potential confounders (*P*_interaction_ = 0.007; Fig. 4). The interaction between BMI and *SLC30A8* genotype also remained after further adjustment for additional dietary confounders (*P*_interaction_ = 0.008; data not tabulated). We observed a significant three-way interaction between the *SLC30A8* genotype, BMI, and the zinc to iron ratio (*P*_interaction_ = 0.007; Fig. 5). A high zinc to iron ratio was strongly associated with a lower risk of T2D among obese subjects with the CT/TT genotype compared to the CC genotype (HR = 0.56, 95% CI 0.42–0.73; *P*_trend_ = 1.5 × 10^−5^; Fig. 5). A non-significant three-way interaction was similarly observed with zinc supplement use, BMI, and *SLC30A8* genotype (*P*_interaction_ = 0.11), in that the combined protective association of the T-allele and zinc supplement use was stronger among obese subjects. No evidence for effect modification by BMI on the *SLC30A8* interaction with total zinc intake or dietary zinc intake on risk of T2D was observed.

## Discussion

In this prospective cohort study, we found that zinc supplement use may confer a protective effect on risk of T2D after adjustment for known and putative confounders. We observed that BMI modified the association between the *SLC30A8* genotype and T2D risk. In addition, a higher zinc to iron ratio was associated with a lower risk of T2D, particularly among overweight and obese subjects with the CT/TT genotypes of *SLC30A8* rs13266634. Surprisingly, a high dietary zinc intake is associated with higher risk of T2D in our study population. After additional adjustment for protein intake, iron intake, and red meat, there was a null association between dietary zinc intake and T2D risk in this study population. This finding may suggest that dietary zinc intake within the normal range does not influence risk of T2D without fully accounting for other nutrients which affect the bioavailability of zinc (e.g., iron).

Two prospective studies in women have previously reported a lower risk of T2D among subjects with a high zinc to iron ratio [6, 7]. In contrast to these studies, we observed no protective effect of dietary zinc intake, but a lower risk of T2D only among zinc supplement users. Zinc supplementation has in randomized trials been shown to lower fasting glucose and HbA1c [8], yet no strong evidence exists showing that this would translate to a lower risk of T2D [9]. In a case-control study, plasma zinc concentration was observed to interact with the *SLC30A8* genotype (rs13266634) on T2D risk, such that the protective effect of higher plasma zinc was stronger among subjects with the TT genotype [15]. Further, in a meta-analysis of 14 cohorts, including the cardiovascular sub-cohort of the MDCS (*N* = 4867), higher total zinc associated with lower fasting glucose levels and this association was stronger among A-allele carriers of rs11558471 *SLC30A8* variant (in strong linkage disequilibrium with rs13266634) [36]. In a genotype-based clinical trial in a healthy Amish population, a 14-day zinc supplementation (50 mg two times per day) resulted in improved early insulin response after an intravenous glucose load among subjects with the CT/TT genotype of rs13266634 [37]. The results from these studies along with the results from our study suggest that zinc interventions may benefit from considering *SLC30A8* rs13266634 genotype. To our knowledge, an interaction between BMI and *SLC30A8* genotype on prospective risk of T2D has not been shown earlier. Our finding that the effect of the risk-increasing CC genotype on T2D risk was stronger among obese subjects may be considered to be in contrast to two previous reports. Timpson et al. reported nominal evidence for a genome-wide effect size heterogeneity for the *SLC30A8* locus, with the rs13266634 SNP showing evidence for an association between the risk allele and reduced BMI when restricted to subjects with T2D [38]. In a study by Cauchi et al., the *SLC30A8* variant was only associated with T2D among non-obese subjects, although there was no significant heterogeneity [39]. It is impossible to deduce from this observational setting whether obesity per se influences the function of the *SLC30A8* gene variant. However, it is plausible to speculate that there may be a synergistic effect of obesity on T2D risk in relation to the *SLC30A8* variant, potentially driven by the combined impact of lower insulin secretion and obesity-associated elevations in glucose levels and insulin resistance. The interaction between BMI, *SLC30A8* genotype, and zinc to iron ratio does suggest that zinc supplementation may have potential to lower risk of T2D among obese subjects and that this effect may be particularly prominent among subjects with the T-allele of rs13266634. Notably, regardless of genotype, zinc supplementation was associated with lower risk of T2D in our population of middle-aged Swedish men and women. The contrasting findings regarding dietary zinc intake in this study compared to a previous study within the Nurses’ Health Study (NHS) cohort [6] could be due to differences in food sources of zinc as well as food sources of other nutrients that may impact the bioavailability of zinc. While the bioavailability of zinc from supplements is higher than from foods, no additional benefit of zinc supplements was found in the NHS as compared to dietary zinc [6]. Similar to differences in food sources of zinc between these cohorts, the type, amount, and consumption pattern of diet supplements may also be very different between populations.

There are several limitations of this study that should be discussed. Although we adjusted for several known and putative lifestyle and dietary confounders, it is not possible to exclude the possibility of residual confounding explaining the observed associations, particularly since there are several dietary factors that have been proposed to influence β-cell function [40]. In addition, isolation of single-nutrient effects in observational settings is difficult due to collinearity between dietary intakes and correlated measurement errors, both which may affect multivariable modeling. A major limitation of this study is that assessment of dietary and supplement zinc use was only assessed at the baseline examinations and relied on self-report. The current study has several notable strengths. To the best of our knowledge, this is the first prospective study examining the potential interaction between *SLC30A8* rs13266634 and zinc intake, from both diet and supplements, on T2D risk. The prospective nature of the study allowed zinc intake assessment prior to development of T2D suggesting that any exposure misclassification is non-differential. The large sample size with virtually no loss to follow-up (< 0.5%) assured that we had sufficient power to detect potential interactions between zinc intakes, measured using a high-validity dietary assessment method, and the *SLC30A8* genetic variant. In addition, we were able to access several national and regional registries in order to identify incident T2D cases, minimizing potential outcome misclassification. Any residual misclassification of T2D status would plausibly attenuate the observed associations and interactions. Since the MDCS is a population-based cohort, the results are also likely to be fairly generalizable to other similar populations [20].

While there is accumulating evidence to suggest that rs13266634 impacts diabetes risk and insulin secretion traits and is affected by total zinc intake and circulating zinc levels, the functional implications of the rs13266634 variant remain unclear [12]. Indeed, a recent study found that rare loss-of-function variants in *SLC30A8* are protective against diabetes [41], which may propose that the rs13266634 C-allele could have some kind of gain-of-function effect. Further, since zinc is an essential component of numerous proteins and exert independent actions, zinc supplementation may have far-reaching effects [3, 42]. As noted previously [17] and based on our results, other nutrients that affect absorption (e.g., iron) or actions of zinc may need to be considered if aiming for individualized prevention or treatment based on the rs13266634 genotype.

## Conclusions

This study provides novel evidence that zinc supplement use and/or a high zinc to iron ratio may be associated with a lower risk of T2D, particularly among subjects with high BMI, and that the effect may be modified by the *SLC30A8* rs13266634 genotype. The findings implicate that when using zinc supplementation for T2D prevention, both obesity status and *SLC30A8* genotype may need to be considered. However, due to the observational nature of our study, the findings should be interpreted with caution and are in need of further confirmation.

## Additional file

Additional file 1: Figure S1. Flow chart of analytical study population. **Figure S2.** Nelson-Aalen cumulative hazard estimates for type 2 diabetes by *SLC30A8* rs13266634 genotype among 20,929 participants in the Malmö Diet and Cancer Study (*P*_logrank_ = 0.0005). Table S1. Baseline characteristics by type 2 diabetes (T2D) status in the Malmö Diet and Cancer Study at baseline (1991–1996). Table S2. Baseline characteristics by *SLC30A8* genotype (rs13266634) in the Malmö Diet and Cancer Study at baseline (1991–1996). (DOCX 10736 kb)

## Abbreviations

BMI: Body mass index
CI: Confidence interval
HR: Hazard ratio
MDCS: Malmö Diet and Cancer Study
SLC30A8: Solute carrier family 30 member 8
T2D: Type 2 diabetes mellitus
