# Supplementary material for: A prospective study of dietary and supplemental zinc intake and risk of type 2 diabetes depending on genetic variation in SLC30A8
Source: Genes Nutr. 2017 Oct 30;12:30. doi: 10.1186/s12263-017-0586-y (PMC5661924; doi:10.1186/s12263-017-0586-y)
Supplement: Additional file 1: Figure S1. — Flow chart of analytical study population. Figure S2. Nelson-Aalen cumulative hazard estimates for type 2 diabetes by SLC30A8 rs13266634 genotype among 20,929 participants in the Malmö Diet and Cancer Study (P logrank = 0.0005). Table S1. Baseline characteristics by type 2 diabetes (T2D) status in the Malmö Diet and Cancer Study at baseline (1991–1996). Table S2. Baseline characteristics by SLC30A8 genotype (rs13266634) in the Malmö Diet and Cancer Study at baseline (1991–1996). (DOCX 10736 kb) [file 12263_2017_586_MOESM1_ESM.docx]

**Additional files**

A Prospective Study of Dietary and Supplemental Zinc Intake and Risk of Type 2 Diabetes Depending on Genetic Variation in *SLC30A8*

Isabel Drake, George Hindy, Ulrika Ericson, Marju Orho-Melander

**Figure S1**. Flow chart of analytical study population.

**Figure S2.** Nelson-Aalen cumulative hazard estimates for type 2 diabetes by *SLC30A8* rs13266634 genotype among 20,929 participants in the Malmö Diet and Cancer Study (P _logrank_ = 0.0005).

**Table S1.** Baseline characteristics by type 2 diabetes (T2D) status in the Malmö Diet and Cancer Study at baseline (1991-1996)

|  | All | No T2D | Incident T2D |
| --- | --- | --- | --- |
| Number of subjects | 26,132 | 22,456 | 3,676 |
| Person-years of follow-up until 31 December 2014 | 455,768 | 415,068 | 40,701 |
|  |  |  |  |
| Total zinc intake (mg/day) | 13.0 (6.1) | 13.0 (6.2) | 12.9 (5.4) |
| Dietary zinc intake (mg/day) | 11.2 (3.3) | 11.1 (3.3) | 11.6 (3.6) |
| Total iron intake (mg/day) | 18.1 (12.1) | 18.1 (12.0) | 18.0 (12.7) |
| Zinc supplement user (%) | 4,417 (16.9) | 3,966 (17.7) | 451 (12.3) |
|  |  |  |  |
| Demography and lifestyle |  |  |  |
| Age (years) | 57.8 (7.6) | 57.8 (7.7) | 58.0 (7.0) |
| Sex (male, %) | 9,874 (37.8) | 8,142 (36.3) | 1,732 (47.1) |
| BMI (kg/m^2^) | 25.6 (3.9) | 25.2 (3.7) | 28.0 (4.3) |
| High physical activity (%) | 6,493 (25.0) | 5,664 (25.4) | 829 (22.8) |
| Current smoker (%) | 7,436 (28.5) | 6,347 (28.3) | 1,089 (29.6) |
| High educational level (%) | 3,807 (14.6) | 3,419 (15.3) | 388 (10.6) |
|  |  |  |  |
| Diet |  |  |  |
| Total energy (kcal) | 2242 (646) | 2236 (641) | 2281 (674) |
| Alcohol (g/day) | 10.8 (12.6) | 10.8 (12.4) | 10.9 (13.5) |
| Dietary fiber (g/day) | 20.0 (7.0) | 20.0 (7.0) | 19.8 (7.0) |
| Processed meat (g/day) | 48.3 (37.3) | 47.6 (37.1) | 52.7 (38.4) |
| Fish and shellfish (g/day) | 45.5 (34.9) | 45.3 (34.6) | 46.4 (36.8) |
| Sugar-sweetened beverages (g/day) | 77.4 (147.6) | 75.8 (145.2) | 87.1 (161.2) |
| Fruit and vegetables (g/day) | 376.2 (185.6) | 377.1 (185.0) | 370.4 (189.5) |
| Coffee (g/day) | 521.2 (396.5) | 522.8 (394.7) | 511.3 (407.1) |
| Diet supplement user (%) | 17,593 (67.3) | 15,097 (67.2) | 2,496 (67.9) |

**Table S2.** Baseline characteristics by *SLC30A8* genotype (rs13266634) in the Malmö Diet and Cancer Study at baseline (1991-1996)

|  | *SLC30A8* genotype | | |
| --- | --- | --- | --- |
|  | CC | CT | TT |
| Number of subjects | 9,621 | 9,123 | 2,185 |
| Person-years of follow-up until 31 December 2014 | 166,903 | 159,169 | 37,935 |
| Number of incident T2D diagnoses | 1,440 | 1,196 | 279 |
|  |  |  |  |
| Total zinc intake (mg/day) | 12.9 (6.2) | 13.0 (5.9) | 12.8 (5.6) |
| Total iron intake (mg/day) | 17.9 (11.7) | 18.3 (12.9) | 18.1 (12.1) |
| Zinc supplement user (n, %) | 1,629 (16.9) | 1,551 (17.0) | 360 (16.5) |
|  |  |  |  |
| Demography and lifestyle |  |  |  |
| Age (years) | 58.0 (7.8) | 58.0 (7.9) | 58.3 (7.9) |
| Sex (male, %) | 3,622 (37.7) | 3,579 (39.2) | 844 (38.6) |
| BMI (kg/m^2^) | 25.6 (3.8) | 25.6 (3.9) | 25.7 (4.1) |
| Height (cm) | 168.6 (8.9) | 168.7 (8.8) | 168.4 (8.8) |
| High physical activity (%) | 2,373 (24.8) | 2,307 (25.4) | 520 (23.9) |
| Current smoker (%) | 2,670 (27.8) | 2,555 (28.0) | 579 (26.5) |
| High educational level (%) | 1,461 (15.2) | 1,314 (14.4) | 310 (14.2) |
|  |  |  |  |
| Diet |  |  |  |
| Total energy (kcal) | 2236 (644) | 2247 (646) | 2243 (644) |
| Alcohol (g/day) | 11.0 (12.6) | 10.9 (12.5) | 10.3 (12.7) |
| Dietary fiber (g/day) | 19.8 (6.9) | 19.9 (7.0) | 19.8 (6.8) |
| Processed meat (g/day) | 48.2 (36.9) | 48.2 (36.8) | 48.9 (39.0) |
| Fish and shellfish (g/day) | 45.6 (35.2) | 45.5 (35.4) | 44.7 (33.9) |
| Sugar-sweetened beverages (g/day) | 77.3 (146.7) | 79.1 (150.2) | 76.6 (140.4) |
| Fruit and vegetables (g/day) | 372.9 (183.4) | 373.9 (182.5) | 369.4 (184.3) |
| Coffee (g/day) | 519.4 (385.9) | 520.0 (399.1) | 503.2 (376.9) |
| Diet supplement user (n, %) | 6,432 (66.9) | 6,154 (67.5) | 1,477 (67.6) |
